# Exploration and application of a highly sensitive bis(salamo)-based fluorescent sensor for B_4_O_7_^2−^ in water-containing systems and living cells

**DOI:** 10.1038/s41598-018-32239-y

**Published:** 2018-09-19

**Authors:** Lu-Mei Pu, Xiao-Yan Li, Jing Hao, Yin-Xia Sun, Yang Zhang, Hai-Tao Long, Wen-Kui Dong

**Affiliations:** 1 College of Science, Gansu Agricultural University, Lanzhou, 730070 China; 2 School of Chemical and Biological Engineering, Lanzhou Jiaotong University, Lanzhou, 730070 China

## Abstract

A highly selective fluorescent sensor H_4_L based on a bis(salamo)-type compound with two N_2_O_2_ chelating moieties as ionophore was successfully developed. Sensor H_4_L was found to have excellent selectivity for B_4_O_7_^2−^ over many other anions (Br^−^, CI^−^, CN^−^, CO_3_^2−^, HCO_3_^−^, H_2_PO_4_^−^, HSO_4_^−^, NO_3_^−^, OAc^−^, S_2_O_3_^−^, SCN^−^, SO_4_^2−^, Hcy (homocysteine) and H_2_O_2_), and it exhibited an approximately 150-fold enhancement of the fluorescence response to B_4_O_7_^2−^ in Tris-HCl buffer (DMF/H_2_O = 9:1, *v*/*v*, pH = 7) solutions. Significantly, its fluorescence intensity was enhanced in a linear fashion with increasing concentrations of B_4_O_7_^2−^. The detection limit of sensor H_4_L towards B_4_O_7_^2−^ was 8.61 × 10^−7^ M. The test strips could conveniently, efficiently and simply detect B_4_O_7_^2−^ ions in Tris-HCl buffer (DMF/H_2_O = 9:1, *v*/*v*, pH = 7) solutions. Furthermore, sensor H_4_L showed excellent membrane permeability in living cells, and it was successfully used to monitor intracellular B_4_O_7_^2−^ by confocal luminescence imaging.

## Introduction

Metal ions and anions both play a key role in daily life^1–4^. Borate, an essential element in the earth, is widely used in industry, agriculture and medicine. For example, borate has widespread use in a solid lubricant in industry, and it may also be applied in welding repair to refrigeration equipment. In medicine, borate could be used for the anti-corrosion of the skin and mucous membranes as well as in the treatment of cancer. In animal medicine, as a feed additive, the research on borate has been attracting increasing attention. Nevertheless, abusing borates not only damages the environment but also endangers human health. Hence, the development of a rapid and convenient detection method for B_4_O_7_^2−^could be of interest.

Up until now, with the development of optical sensors for recognizing heavy and transition metal ions in living organisms^5–15^, intense efforts have been devoted to the design and synthesis of high sensitivity fluorescent sensors due to their low cost and rapid response as well as the easy operability of the fluorescent technique^16–22^. According to the relevant literature, the metal complexes of N_2_O_2_ salen-type ligands and corresponding analogues could be used in catalysis^23,24^, nonlinear optical materials and magnetic materials^25–34^, supramolecular architecture^35,36^, ion recognition^37–45^, biological fields and so forth^46–52^. Today, studies on the participation of salamo-type compounds in ion recognition have yet to be explored^53–63^. Notably, compared with most of the known fluorescent probes for Zn^2+^, Cu^2+^, and CN^−^, there are relatively few reports on fluorescent probes for B_4_O_7_^2−^.

Herein, we have designed and synthesized a bis(salamo)-type sensor H_4_L for the recognition of B_4_O_7_^2−^ in Tris-HCl buffer (DMF/H_2_O = 9:1, *v*/*v*, pH = 7) solutions. The UV–vis absorption spectra and fluorescence titration experiments for sensor H_4_L were investigated and the results indicated that sensor H_4_L has a high selectivity for B_4_O_7_^2−^ over many other ions based on the change in color visible to the naked eye and the fluorescence intensity at a low concentration as well as a mild environment.

## Results and Discussion

### The selectivity of sensor H_4_L to B_4_O_7_^2−^

A series of host-guest recognition experiments were carried out to investigate the B_4_O_7_^2−^ recognition ability of sensor H_4_L with various anions and some compounds, B_4_O_7_^2−^, Br^−^, CI^−^, CN^−^, CO_3_^2−^, HCO_3_^−^, H_2_PO_4_^−^, HSO_4_^−^, NO_3_^−^, OAc^−^, S_2_O_3_^−^, SCN^−^, SO_4_^2−^, Hcy and H_2_O_2_ in Tris-HCl buffer (DMF/H_2_O = 9:1, *v*/*v*, pH 7) solutions. As shown in Fig. S2a, all of the examined anions show the same absorption peaks with sensor H_4_L, however, only the addition of B_4_O_7_^2−^ displayed the highest absorbance under the same reaction conditions. There are no isosbestic points due to the differences in binding abilities between sensor H_4_L and all of these anions.

The interaction of sensor H_4_L and B_4_O_7_^2−^ was evaluated by a UV–vis titration method. As shown in Fig. S2b, with increasing concentrations of B_4_O_7_^2−^ (0.001 M) from 0.0–39.0 equiv. in Tris-HCl buffer (DMF/H_2_O = 9:1, *v*/*v*, pH = 7) solutions, the absorbance showed a linear increase when the ratio of [B_4_O_7_^2−^]/[H_4_L] is below 39:1, and the absorbance no longer changes when the ratio reached 39:1.

### Effect of the pH on sensor H_4_L

**Figure 1:**
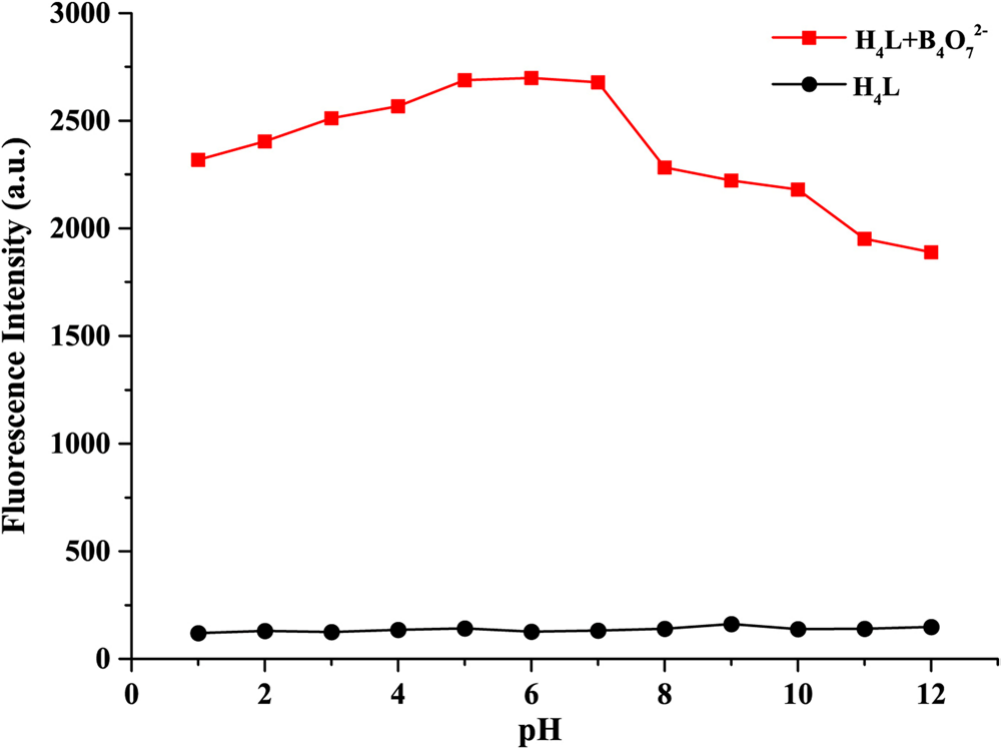
Changes in the fluorescence spectra of H_4_L-B_4_O_7_^2−^ at various pH values at room temperature.

In order to remove the interference by protons during the detection of B_4_O_7_^2−^ and to find the optimal sensing conditions, further tested was performed in the pH range of 1 to 12. As shown in Fig. 1, the results obtained show no dramatic spectral changes of sensor H_4_L in the wide pH range of 1–12, suggesting that sensor H_4_L was very stable. The H_4_L-B_4_O_7_^2−^ displayed a strong fluorescence intensity in the pH range of 1–7. The results above clearly indicate that sensor H_4_L can be employed as a sensitive relay-sensor to recognize and distinguish B_4_O_7_^2−^ in a wide pH range.

### Fluorescence detection of sensor H_4_L towards B_4_O_7_^2−^

**Figure 2:**
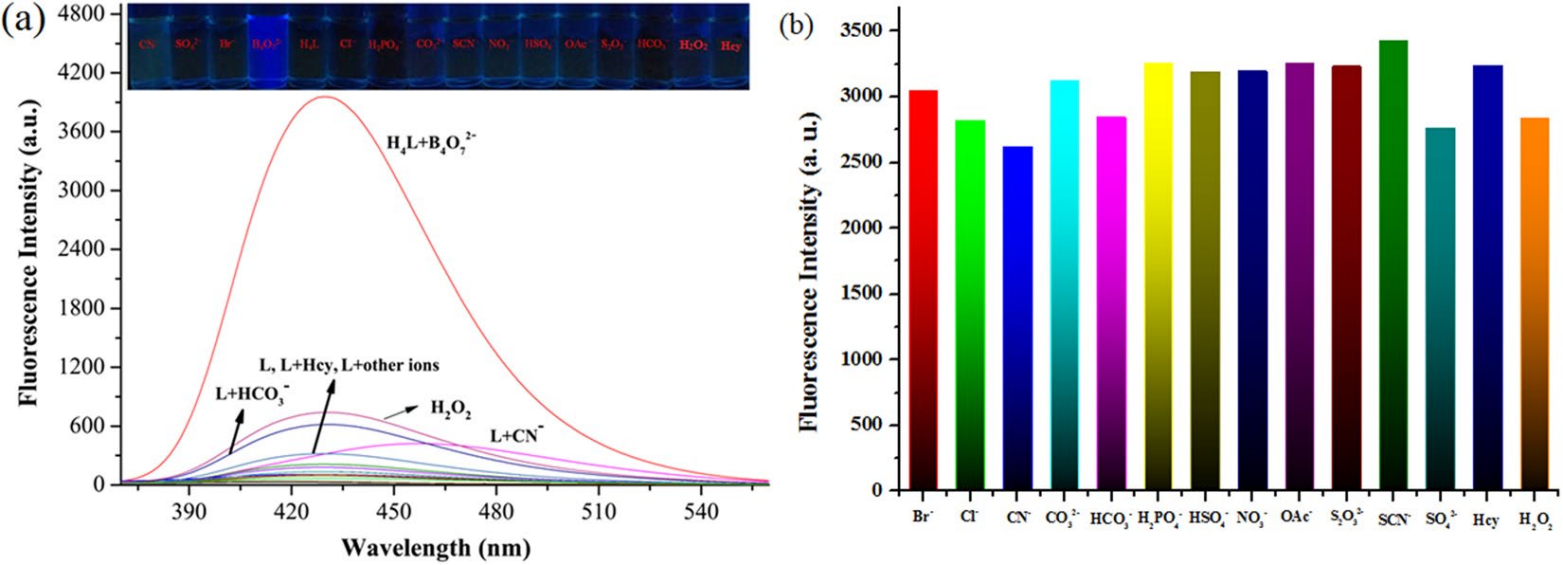
(**a**) Fluorescence spectra and (**b**) fluorescent intensity at 323 nm of sensor H_4_L (0.01 mM) in the presence of various anions (40.0 equiv. of Br^−^, CI^−^, CN^−^, CO_3_^2−^, HCO_3_^−^, H_2_PO_4_^−^, HSO_4_^−^, NO_3_^−^, OAc^−^, S_2_O_3_^−^, SCN^−^, SO_4_^2−^, Hcy and H_2_O_2_) in Tris-HCl buffer (DMF/H_2_O = 9:1, *v*/*v*, pH = 7).

Selectivity is a very important parameter to evaluate the performance of a fluorescence chemosensor. The fluorescence emission spectral responses of sensor H_4_L to various anions and some compounds (B_4_O_7_^2−^, Br^−^, CI^−^, CN^−^, CO_3_^2−^, HCO_3_^−^, H_2_PO_4_^−^, HSO_4_^−^, NO_3_^−^, OAc^−^, S_2_O_3_^−^, SCN^−^, SO_4_^2−^, Hcy and H_2_O_2_) were evaluated in Tris-HCl buffer (DMF/H_2_O = 9:1, *v*/*v*, pH 7) solutions. As shown in Fig. 2a, all of the examined anions did not display any obvious response to sensor H_4_L, and only after the addition of B_4_O_7_^2−^ did, sensor H_4_L produce a significant enhancement of the fluorescence intensity at 430 nm (λ_ex_ = 323 nm). These results suggested that sensor H_4_L displayed an excellent selectivity for B_4_O_7_^2−^ over all of the other anions tested.

To further explore the high selectivity of sensor H_4_L for B_4_O_7_^2−^ in practice, we also investigated the ability of sensor H_4_L to detect B_4_O_7_^2−^ in the presence of equivalent and excess amounts of other anions, to determine whether they would interfere with coordination between sensor H_4_L and B_4_O_7_^2−^. As shown in Fig. 2b, when anions and some compounds, including Br^−^, CI^−^, CN^−^, CO_3_^2−^, HCO_3_^−^, H_2_PO_4_^−^, HSO_4_^−^, NO_3_^−^, OAc^−^, S_2_O_3_^−^, SCN^−^ and SO_4_^2−^, Hcy and H_2_O_2_, were separately added into a mixed solution of sensor H_4_L and B_4_O_7_^2−^, the fluorescence intensity had little or negligible change. Hence, fluorescence interference experiments of various anions revealed that other anions could not affect the sensing process of sensor H_4_L for B_4_O_7_^2−^. In order to further understand the binding behavior of H_4_L with B_4_O_7_^2−^, the ^1^H NMR spectra experiments of H_4_L and H_4_L-B_4_O_7_^2−^ were also performed in DMSO-*d*_6_. The phenolic O-H in H_4_L has completely disappeared upon the addition of B_4_O_7_^2−^, and all protons of the aromatic ring and aldimine CH=N in H_4_L were shifted down-field (Fig. S3). These changes may be due to the destruction of intermolecular electrostatic and hydrogen-bond interactions after the addition of B_4_O_7_^2−^ to H_4_L.

The fluorescence enhancement of the sensor H_4_L response to B_4_O_7_^2−^ may be attributed to that borates are hydrolyzed to form boric acid:

$${[{{\rm{B}}}_{4}{{\rm{O}}}_{5}{({\rm{OH}})}_{4}]}^{2-}+5{{\rm{H}}}_{2}{\rm{O}}\rightleftharpoons 4{{\rm{H}}}_{3}{{\rm{BO}}}_{3}+2{{\rm{OH}}}^{-}\rightleftharpoons 2{{\rm{H}}}_{3}{{\rm{BO}}}_{3}+2{\rm{B}}{({\rm{OH}})}_{4}^{-}$$

Four coordinated organoboron compounds based on N,O-chelation are constructed mainly by structures 1, 2 and 3 as the ligand backbone (Fig. S4a). The weak fluorescence of sensor H_4_L was attributed to the lone pairs of electrons on the nitrogen atoms, which lead to intra-molecular photoinduced electron transfer (PET). Due to the lack of electronic properties, the Lewis bases such as the N atoms of the salamo moieties from the H_4_L unit coordinate to the B atoms, resulting in a unique electronic structure and optical properties after B atoms are incorporated into the conjugated system. Four coordinated organoboron compounds can produce strong fluorescence with the excitation of light^64^. On the other hand, sensor H_4_L exhibited a very weak fluorescence intensity due to the photoinduced electron transfer process from the hydroxy oxygen atom to amino groups. However, when sensor H_4_L was coordinated with a B_4_O_7_^2−^ ion, the chelation-enhanced fluorescence process would be started, and the photoinduced electron transfer process would be inhibited at the same time (Fig. S4b). Hence, an obvious enhancement of the fluorescence intensity was observed.

**Figure 3:**
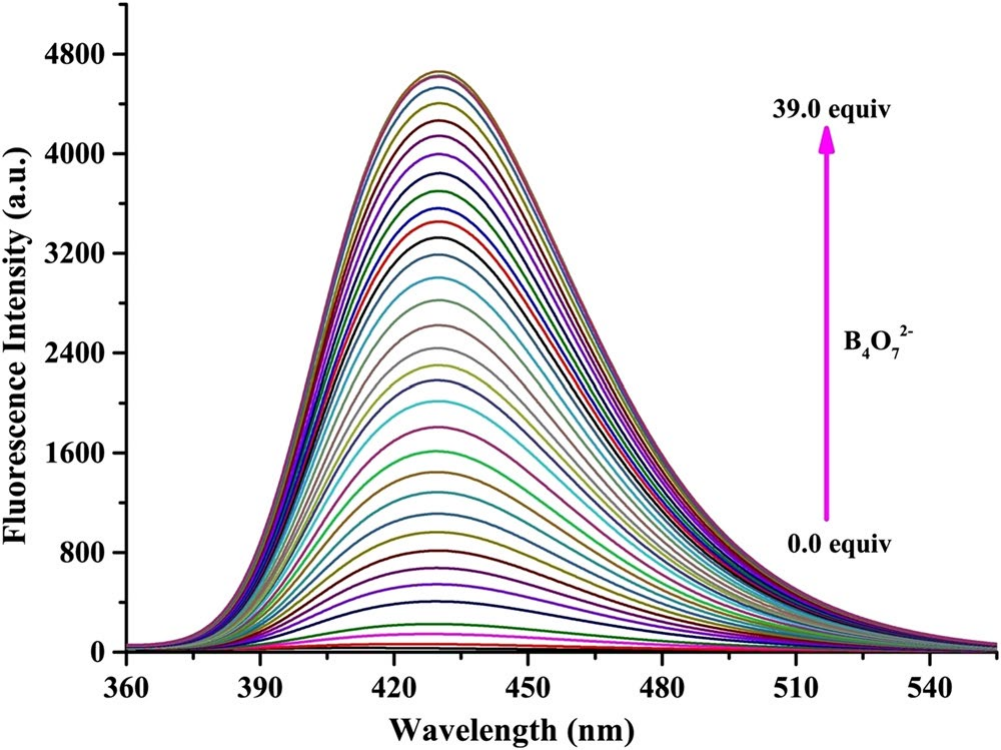
Fluorescence emission spectra of sensor H_4_L (0.01 mM) upon the subsequent addition of B_4_O_7_^2−^ (0–39 equiv. λ_ex_ = 323 nm) in Tris-HCl buffer (DMF/H_2_O = 9:1, *v*/*v*, pH = 7) solutions.

Fluorescent titration was carried out to gain more insight into the recognition properties of sensor H_4_L as a B_4_O_7_^2−^ probe. As shown in Fig. 3, without B_4_O_7_^2−^, sensor H_4_L had nearly no fluorescence. However, with increasing concentrations of B_4_O_7_^2−^, the fluorescence intensity was remarkably increased at 430 nm. Significantly, a good linear relationship between the fluorescence intensity and the B_4_O_7_^2−^ concentration could be obtained (R^2^ = 0.95873), which is based on the fluorescence titration experiment. It can be seen that the fluorescence intensity change was nearly linear with the increase of concentration of B_4_O_7_^2−^ (Fig. S5). For many practical applications, it is very meaningful to detect the analytes at low concentrations. Meanwhile, based on the corrected Benesi-Hildebrand formula, the binding constant for the binding of B_4_O_7_^2−^ to sensor H_4_L was calculated as 4.72 × 10^3^ M^−1^ ^65,66^. The detection limit (LOD) could be calculated to be 8.61 × 10^−7^ M and the limit of quantitation (LOQ = 2.87 × 10^−6^ M) of sensor H_4_L for B_4_O_7_^2−^ anions was also obtained^67^. The LOD and LOQ were calculated based on the following equations:

$${\rm{LOD}}=3\times {\rm{\delta }}/{\rm{S}};\,{\rm{LOQ}}=10\times {\rm{\delta }}/{\rm{S}}.$$

Where δ (δ = 3.9 × 10^−5^) represents the standard deviation of the blank measurements, and S is the slope of the intensity versus sample concentration curve^68,69^.

**Figure 4:**
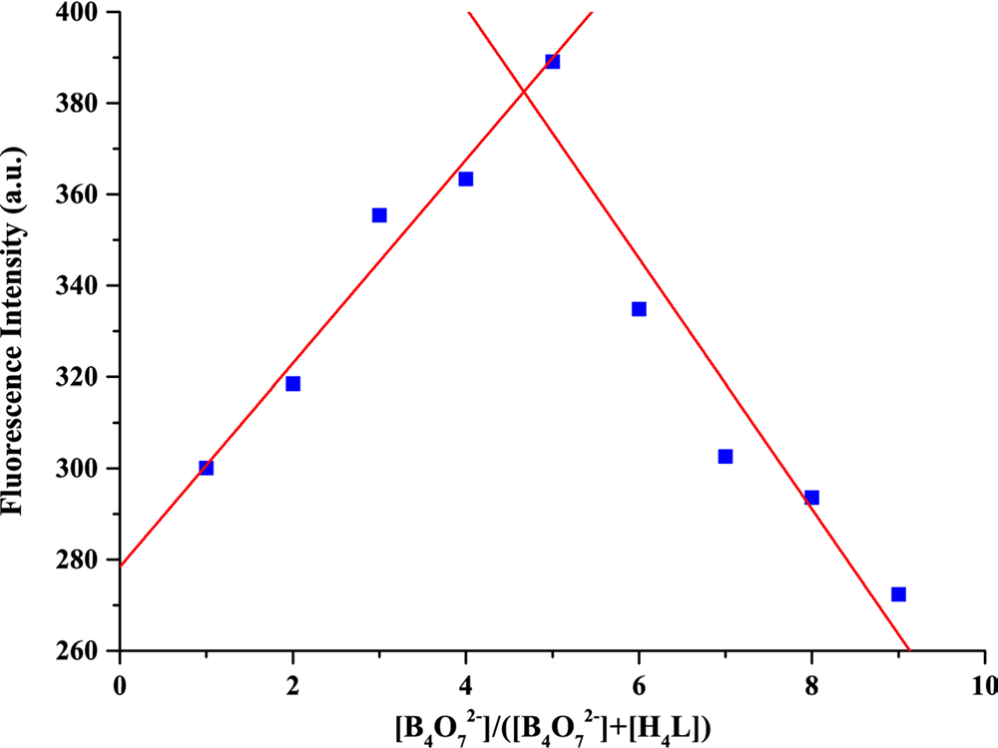
Job’s plot for determining the stoichiometry of sensor H_4_L and B_4_O_7_^2−^ in Tris-HCl buffer (DMF/H_2_O = 9:1, *v*/*v*, pH = 7). Excitation wavelength: 323 nm.

We investigated the binding stoichiometry and binding affinities of sensor H_4_L and B_4_O_7_^2−^. A Job’s plot analysis for the fluorescence intensity was also measured by keeping the sum of the initial concentrations of sensor H_4_L and B_4_O_7_^2−^ constant at 10 µM (Fig. 4). The experiment was performed in Tris-HCl buffer (DMF/H_2_O = 9:1, *v*/*v*, pH = 7) solutions at an excitation wavelengths of 323 nm. The results indicated that the binding stoichiometry between sensor H_4_L and B_4_O_7_^2−^ is 1:1.

**Figure 5:**
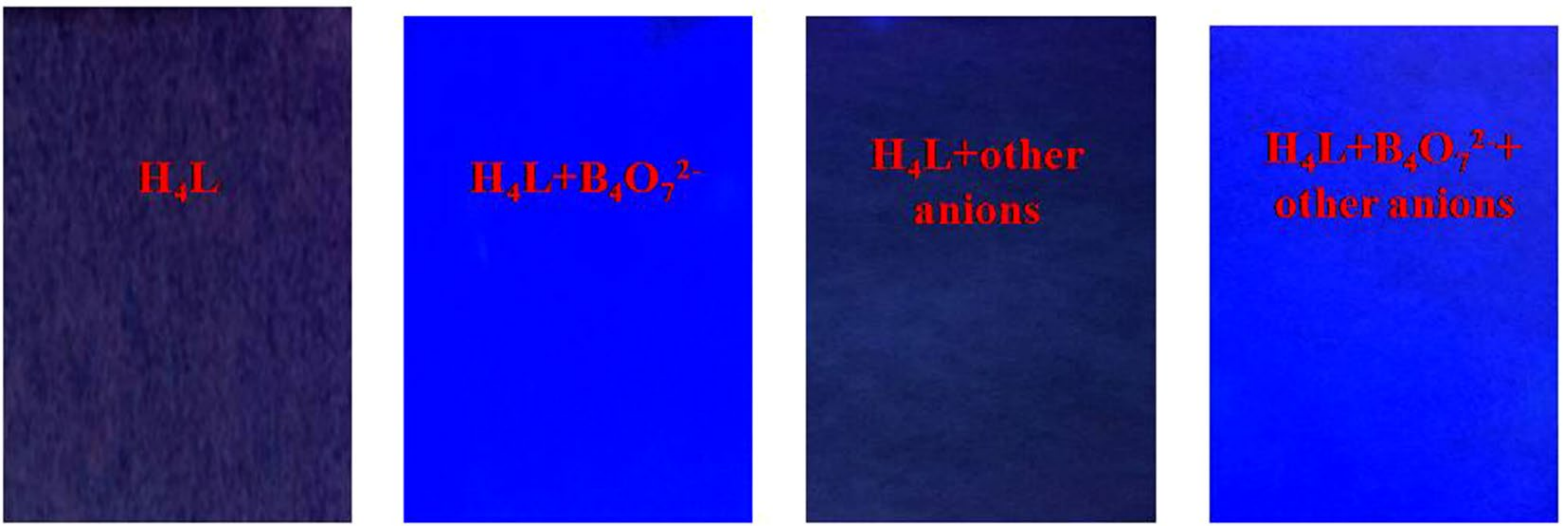
Photographs of the colorimetric test kit with H_4_L for detecting B_4_O_7_^2−^ under irradiation at 365 nm.

The realization of a quick response to B_4_O_7_^2−^ is very meaningful for sensor H_4_L in its practical application in portable sensing devices. To facilitate the use of sensor H_4_L for the detection of B_4_O_7_^2−^, test strips were made by soaking filter papers in a Tris-HCl buffer (DMF/H_2_O = 9:1, *v*/*v*, pH = 7) solution of sensor H_4_L followed by exposure to air until complete drying. Intriguingly, the obvious fluorescence color changes were observed immediately from gray to light blue in visible light when B_4_O_7_^2−^ anions were added. Therefore, sensor H_4_L exhibited excellent fluorescence sensing performance, which would be very useful for the fabrication of sensing devices with fast and convenient detection of B_4_O_7_^2−^ (Fig. 5).

In order to be applied in real life and to find the optimal sensing conditions, the fluorescence intensity of sensor H_4_L over a period of time in the presence of B_4_O_7_^2−^ was determined in Tris-HCl buffer (DMF/H_2_O = 9:1, *v*/*v*, pH = 7) solutions. As shown in Fig. S6a, it was found that there were nearly no changes in the fluorescence intensity of H_4_L-B_4_O_7_^2−^ over a period of time, suggesting that H_4_L-B_4_O_7_^2−^ was very stable. Additionally, the fluorescence intensities at different temperatures were also determined. As shown in Fig. S6b, H_4_L exhibited satisfactory B_4_O_7_^2−^ sensing abilities when the temperature was in the range of 0–90 °C. Therefore, it was demonstrated that sensor H_4_L could work in a short time and at room temperature, and it can be applied in real life.

**Figure 6:**
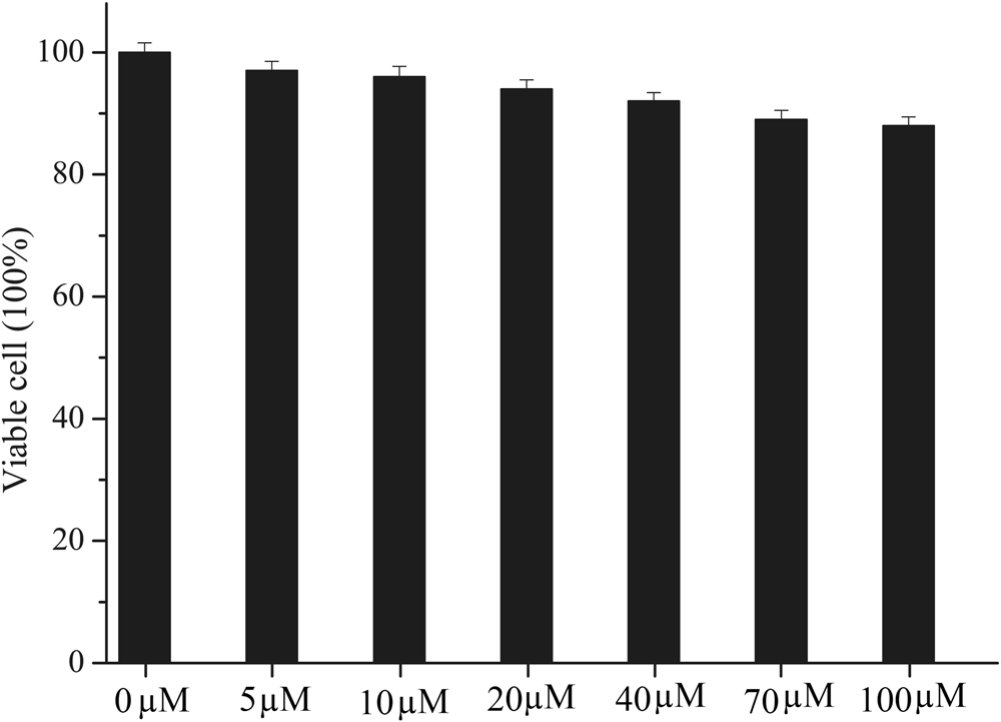
Cytotoxicity assays of H_4_L at different concentrations for BHK-21 cells.

**Figure 7:**
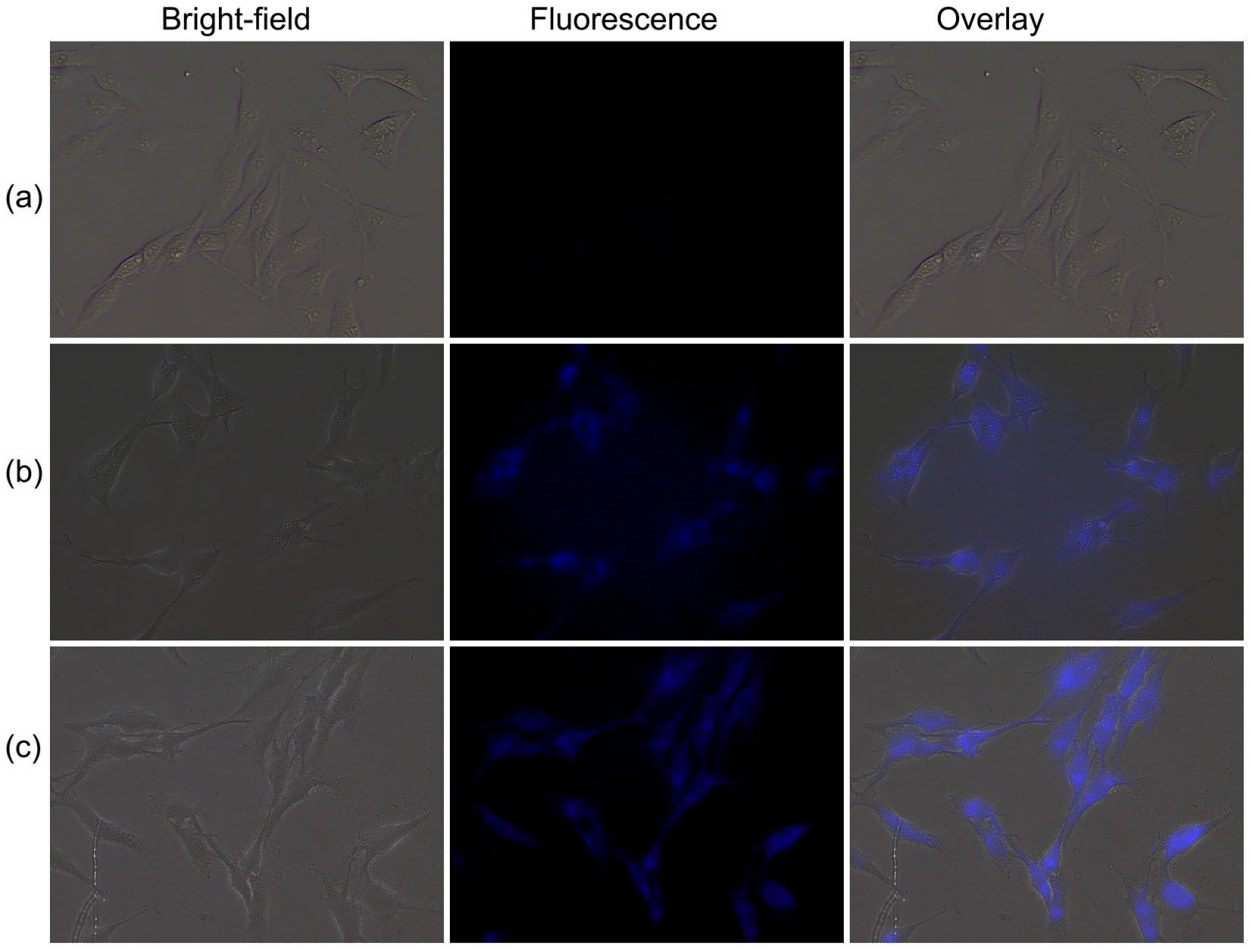
Confocal luminescence images of BHK-21 cells. (**a**) BHK-21 cells were incubated with sensor H_4_L (30 μM) for 30 min at 37 °C and (**b**) then further incubated with B_4_O_7_^2−^ (100 μM) for 30 min.

Prior to the imaging experiments, the cytotoxicity of H_4_L at different concentrations (0–100 µM) was evaluated through 3-(4,5-dimethylthiazol-2-yl)-2,5-diphenyltetrazolium bromide (MTT) assays in BHK-21 cells. The results after 48 h revealed that H_4_L exhibited almost no toxicity or low toxicity (Fig. 6).The ability of sensor H_4_L to detect B_4_O_7_^2−^ in living cells was further studied by confocal luminescence imaging. As seen in Fig. 7, the BHK-21 cells incubated with sensor H_4_L (30 μM) alone for 30 min at 37 °C maintained a good shape and were viable, the solvent for the H_4_L concentrate is DMSO, and they also showed very good intracellular fluorescence. Interestingly, an enhanced intracellular fluorescence was detected in cells containing sensor H_4_L incubated with B_4_O_7_^2−^ for 3 h. From confocal fluorescence images of the BHK-21 cells, it was revealed that sensor H_4_L displayed good cell permeability and could be used to detect B_4_O_7_^2−^ ions in living cells.

In conclusion, we designed and synthesized a bis(salamo)-type sensor H_4_L, which showed excellent recognition of B_4_O_7_^2−^with different fluorescence changes and changes in color. In addition, the detection limit of the fluorescence response of sensor H_4_L to B_4_O_7_^2−^ is as low as 8.61 × 10^−7^ M. This sensing system shows many advantages. The test strips could conveniently, low cytotoxicity, efficiently and simply detect B_4_O_7_^2−^ in solutions. In addition, the free sensor H_4_L was achieved through regeneration by using EDTA and was able to further sense B_4_O_7_^2−^. We believe that this study provides a potential application for constructing a fluorescent sensor for the highly sensitive and rapidly recognition of B_4_O_7_^2−^ ions based on different fluorescence intensities and changes in color in practical life.

## Materials and General Methods

2-Hydroxy-3-methoxybenzaldehyde (99%), methyl trioctyl ammonium chloride (90%), pyridiniumchlorochromate (98%) and borontribromide (99.9%) were purchased from Alfa Aesar. Hydrobromic acid 33 wt% solution in acetic acid was purchased from J&K Scientific Ltd. The other reagents and solvents were analytical grade reagents from the Tianjin Chemical Reagent Factory and were used as received. Melting points were obtained by the use of a microscopic melting point apparatus made by the Beijing Taike Instrument Limited Company and were uncorrected. ^1^H NMR spectra was determined by a German Bruker AVANCE DRX-400 spectrophotometer. All of the UV–vis and fluorescence spectroscopy experiments were recorded on Shimadzu UV-2550 and Perkin-Elmer LS-55 spectrometers, respectively.

### Synthesis of sensor H_4_L

**Figure 8:**
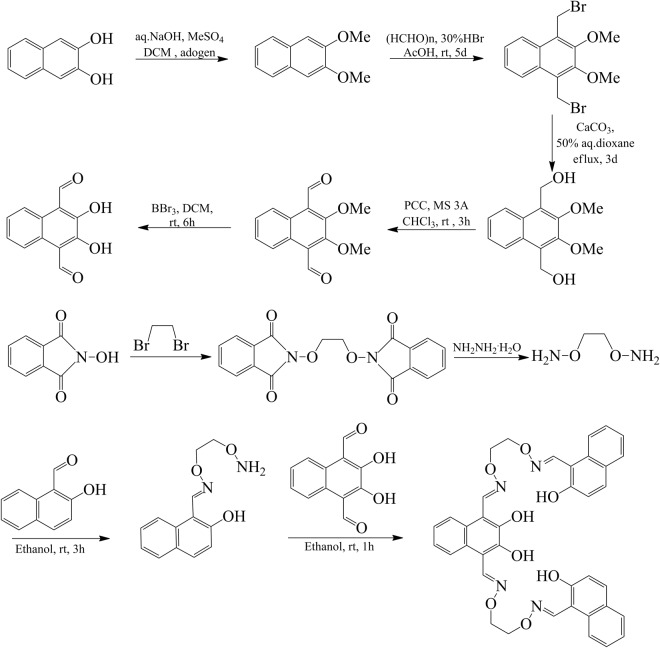
Synthetic route to sensor H_4_L.

The bis(salamo)-type sensor H_4_L was synthesized according to the previously reported procedure^70–78^. The IR, ^1^H NMR and UV-vis spectra of H_4_L are nearly consistent with the literature data (Fig. S1). The major reaction steps of sensor H_4_L are demonstrated in Fig. 8.

### Statistical analysis

Statistical methods used are detailed at each experiment individually.

## Electronic supplementary material

Supplementary Material

## Data Availability

The datasets generated during and/or analysed during the current study are available from the corresponding author on reasonable request.

## References

[1] Chai LQ (2017). Structural, spectral, electrochemical and DFT studies of two mononuclear manganese(II) and zinc(II) complexes. Polyhedron.

[2] Chai LQ (2017). Two mono- and dinuclear Ni(II) complexes constructed from quinazoline-type ligands: synthesis, X-ray structures, spectroscopic, electrochemical, thermal, and antimicrobial studies. Polyhedron.

[3] Chai LQ (2016). Synthesis, crystal structure, spectroscopic properties and DFT calculations of a new schiff base-type Zinc(II) complex. Res. Chem. Intermed..

[4] Chai LQ (2016). Synthesis, x-ray structure, spectroscopic, electrochemical properties and DFT calculation of a bridged dinuclear copper(II) complex. Inorg. Chim. Acta.

[5] Bazzicalupi C (2012). Thermodynamic and fluorescence emission properties of the zn(II), cd(II) and pb(II) complexes with a fluorescent chelator bearing phenanthroline and naphthalene subunits. Inorg. Chim. Acta.

[6] Khania H (2010). Multi-walled carbon nanotubes-ionic liquid-carbon paste electrode as a super selectivity sensor: application to potentiometric monitoring of mercury ion(II). J. Hazard. Mater.

[7] Gupta VK (2011). Recent advances on potentiometric membrane sensors for pharmaceutical analysis. Comb. Chem. High Tscr..

[8] Gupta VK, Jain AK, Maheshwari G (2007). Aluminum(III) selective potentiometric sensor based on morin in poly(vinyl chloride) matrix. Talanta.

[9] Gupta VK (2011). Agarwal, Electrochemical analysis of some toxic metals by ion–selective electrodes. Crit. Rev. Anal. Chem..

[10] Gupta VK (2007). An iron(III) ion-selective sensor based on a μ-bis(tridentate) ligand. Talanta.

[11] Zhu BC (2018). A highly specific and ultrasensitive near-infrared fluorescent probe for imaging basal hypochlorite in the mitochondria of living cells. Biosens. Bioelectron..

[12] Wang YW (2017). A highly specific and ultrasensitive fluorescent probe for basal lysosomal HOCl detection based on chlorination induced by chlorinium ions (Cl^+^). J. Mater. Chem. B.

[13] Zhu BC (2018). A fast-response, highly specific fluorescent probe for the detection of picomolar hypochlorous acid and its bioimaging applications. Sens. Actuators B.

[14] Zhu BC (2018). A highly specific far-red fluorescent probe for imaging endogenous peroxynitrite in the mitochondria of living cells. Sens. Actuators B.

[15] Zhu BC (2018). A highly selective and ultrasensitive ratiometric far-red fluorescent probe for imaging endogenous peroxynitrite in living cells. Sens. Actuators B.

[16] Gupta VK (2006). Copper(II)-selective potentiometric sensors based on porphyrins in PVC matrix. Sens. Actuators B.

[17] Gupta VK, Chandra S, Lang H (2005). A highly selective mercury electrode based on a diamine donor ligand. Talanta.

[18] Gupta VK (2013). Mercury selective potentiometric sensor based on low rim functionalized thiacalix [4]-arene as a cationic receptor. J. Mol. Liq..

[19] Xu Z (2011). A highly sensitive and selective colorimetric and off–on fluorescent chemosensor for Cu^2+^ based on rhodamine B derivative. Sens. Actuators B.

[20] Jun ME, Roy B, Ahn KH (2011). **“**Turn-on” fluorescent sensing with “reactive” probes. Chem. Commun..

[21] Du J (2012). Fluorescent chemodosimeters using “mild” chemical events for the detection of small anions and cations in biological and environmental media. Chem. Soc. Rev..

[22] Chen X (2012). Fluorescent chemosensors based on spiroring-opening of xanthenes and related derivatives. Chem. Rev..

[23] Li XY (2017). Syntheses, crystal structures and catalytic activities of two solvent-induced homotrinuclear co(II) complexes with a naphthalenediol-based bis(Salamo)-type tetraoxime ligand. RSC Adv..

[24] Li, L. H. *et al*. Syntheses, structures and catecholase activities of homo- and hetero-trinuclear cobalt(II) complexes constructed from an acyclic naphthalenediol-based bis(salamo)-type ligand, 10.1002/aoc.3818.

[25] Wang L (2016). A Novel self-assembled nickel(II)-cerium(III) heterotetranuclear dimer constructed from N_2_O_2_-type bisoxime and terephthalic acid: synthesis, structure, and photophysical properties. Z. Anorg. Allg. Chem..

[26] Ma JC (2016). An unexpected dinuclear Cu(II) complex with a bis(salamo) chelating ligand: synthesis, crystal structure, and photophysical properties. J. Coord. Chem..

[27] Tao CH (2017). Heterobimetallic 3d–4f Zn(II)–Ln(III) (Ln = Sm, Eu, Tb and Dy) complexes with a N_2_O_4_ bisoxime chelate ligand and a simple auxiliary ligand Py: syntheses, structures and luminescence properties. Polyhedron.

[28] Dong YJ (2017). Three asymmetric salamo-type copper(II) and cobalt(II) complexes: syntheses, structures and fluorescent properties. Polyhedron.

[29] Dong YJ (2017). Four 3d–4f heteromultinuclear zinc(II)–lanthanide(III) complexes constructed from a distinct hexadentate N_2_O_2_-type ligand: syntheses, structures and luminescence properties. J. Coord. Chem..

[30] Song XQ (2016). Two dodecanuclear heterometallic [Zn_6_Ln_6_] clusters constructed by a multidentate salicylamide salen-like ligand: synthesis, structure, luminescence and magnetic properties. Dalton Trans..

[31] Liu PP (2015). Synthesis, structure and magnetic properties of a new one dimensional manganese coordination polymer constructed by a new asymmetrical ligand. Inorg. Chim. Acta.

[32] Song XQ (2015). Four polynuclear complexes based on a versatile salicylamide salen-like ligand: synthesis, structural variations and magnetic properties. Inorg. Chim. Acta.

[33] Song XQ (2015). Substituted group-directed assembly of Zn(II) coordination complexes based on two new structural related pyrazolone based salen ligands: syntheses, structures and fluorescence properties. Inorg. Chim. Acta.

[34] Liu PP (2017). Pentanuclear sandwich-type Zn^II^-Ln^III^ clusters based on a new salen-like salicylamide ligand: structure, near-infrared emission and magnetic properties. Polyhedron.

[35] Wang P (2015). Synthesis, structure and spectroscopic properties of the trinuclear cobalt(II) and nickel(II) complexes based on 2-hydroxynaphthaldehyde and bis(aminooxy)alkane. Spectrochim. Acta A.

[36] Wang P (2016). L. An infinite 2D supramolecular cobalt(II) complex based on an asymmetric salamo-type ligand: synthesis, crystal structure, and spectral properties. Synth. React. Inorg., Met.- Org., Nano-Met. Chem..

[37] Hu JH (2015). Selective colorimetric and “turn-on” fluorimetric detection of cyanide using an acylhydrazone sensor in aqueous media. New J. Chem..

[38] Hu JH (2015). Highly selective and effective mercury(II) fluorescent sensor. New J. Chem..

[39] Li JB (2014). Cyanide detection using a benzimidazole derivative in aqueous media. Spectrochim. Acta A.

[40] Hu JH (2015). Acylhydrazone based fluorescent chemosensor for zinc in aqueous solution with high selectivity and sensitivity. Sens. Actuators B.

[41] Hu JH (2014). A cyanide ion probe based on azosalicylic aldehyde of benzoyl hydrazone, *Chinese J*. Inorg. Chem..

[42] Qi J (2016). Cyanide detection using azo-acylhydrazone in aqueous media with high sensitivity and selectivity. Current Anal. Chem..

[43] Hu JH (2016). Studies on the crystal structure and characterization of N-(4-acetylphenyl)-N’-(2-nitrobenzoyl)-thiourea, phosphorus. sulfur, Silicon Relat. Elem..

[44] Hu JH (2016). A colorimetric and “turn-on” fluorimetric chemosensor for the selective detection of cyanide and its application in food samples. RSC Adv..

[45] Hu JH (2017). A new unsymmetrical azine derivative based on coumarin group as dual-modal sensor for CN^−^ and fluorescent “OFF–ON” for Zn^2+^. Spectrochim. Acta A..

[46] Wu HL (2014). Study on synthesis, crystal structure, antioxidant and DNA-binding of mono-, di- and poly-nuclear lanthanides complexes with bis(*N*-salicylidene)-3-oxapentane-1,5-diamine. J. Photochem. Photobiol. B.

[47] Wu HL (2014). Synthesis, crystal structure, antioxidation and DNA-binding properties of a dinuclear copper(II) complex with bis(*N*-salicylidene)-3-oxapentane-1,5-diamine. J. Coord. Chem..

[48] Wu HL (2015). Synthesis, structure, antioxidation, and DNA-binding studies of a binuclear ytterbium(III) complex with bis(*N*-salicylidene)-3-oxapentane-1,5-diamine. Res. Chem. Intermed..

[49] Wu HL (2015). A new manganese(III) complex from bis(5-methylsalicylaldehyde)-3-oxapentane-1,5-diamine: synthesis, characterization, antioxidant activity and luminescence. J. Chin. Chem. Soc..

[50] Chen CY (2015). Gadolinium(III) and dysprosium(III) complexes with a schiff base bis(*N*-salicylidene)-3-oxapentane-1,5-diamine: synthesis, characterization, antioxidation, and DNA-binding studies. J. Coord. Chem..

[51] Wu HL (2014). Two Lanthanide(III) complexes based on the schiff base *N*,*N*′-Bis(salicylidene)-1,5-diamino-3-oxapentane: synthesis, characterization, DNA-binding properties, and antioxidation. Z. Anorg. Allg. Chem..

[52] Wu HL (2014). Preparation, structure, DNA-binding properties, and antioxidant activities of a homodinuclear erbium(III) complex with a pentadentate schiff base ligand. J. Chem. Res..

[53] Wang BJ (2017). A novel relay-sensor for highly sensitive and selective detection of Zn^2+^/Pic^−^ and fluorescence on/off switch response of H^+^/OH^−^. Sens. Actuators B.

[54] Dong YJ (2017). A highly selective visual and fluorescent sensor for Pb^2+^ and Zn^2+^ and crystal structure of Cu^2+^ complex based-on a novel single-armed salamo-type bisoxime. Supramol. Chem..

[55] Dong WK (2016). Four new nickel(II) complexes based on an asymmetric salamo-type ligand: synthesis, structure, solvent effect and electrochemical property. Inorg. Chim. Acta.

[56] Dong WK (2016). Salamo-type trinuclear and tetranuclear cobalt(II) complexes based on a new asymmetry salamo-type ligand: syntheses, crystal structures, and fluorescence properties. J. Coord. Chem..

[57] Dong WK (2016). A series of heteromultinuclear zinc(II)–lanthanide(III) complexes based on 3-MeOsalamo: syntheses, structural characterizations, and luminescent properties. Cryst. Growth Des..

[58] Dong XY (2017). A dinuclear nickel(II) complex derived from an asymmetric salamo-type N_2_O_2_ chelate ligand: synthesis, structure and optical properties. Z. Naturforsch..

[59] Dong WK (2016). Construction of mononuclear copper(II) and trinuclear cobalt(II) complexes based on asymmetric salamo-type ligands. Z. Anorg. Allg. Chem..

[60] Dong WK (2016). A new application of salamo-type bisoximes: as a relay–sensor for Zn^2+^/Cu^2+^ and its novel complexes for successive sensing of H^+^/OH^−^. Sens. Actuators B.

[61] Dong WK (2016). Novel multinuclear transition metal(II) complexes based on an asymmetric salamo-type ligand: syntheses, structure characterizations and fluorescent properties. Inorg. Chim. Acta.

[62] Dong WK (2016). Di- and tetranuclear heterometallic 3d–4f cobalt(II)–lanthanide(III) complexes derived from a hexadentate bisoxime: syntheses, structures and magnetic properties. Polyhedron.

[63] Dong WK (2016). Nine self-assembled nickel(II)–lanthanide(III) heterometallic complexes constructed from a salamo-type bisoxime and bearing a N- or O-donor auxiliary ligand: syntheses, structures and magnetic properties. New J. Chem..

[64] Qin YY (2017). Four coordinated organoboron compounds with π-conjugated N,O-chelate ligand and their optoelectronic applications, *Chinese J*. Inorg. Chem..

[65] Huang WB (2017). A porphyrin-based fluorescent probe for optical detection of toxic Cd^2+^ ion in aqueous solution and living cells. Dyes and Pigments.

[66] Xu L (2013). A simple fluorescent probe for Cd^2+^ in aqueous solution with high selectivity and sensitivity. Dalton Trans..

[67] Liu ZP (2010). A highly sensitive ratiometric fluorescent probe for Cd^2+^ detection in aqueous solution and living cells. Chem. Commun..

[68] Wang P (2015). A novel peptide-based fluorescent chemosensor for measuring zinc ions using different excitation wavelengths and application in live cell imaging. J. Mater. Chem. B.

[69] Ru JX (2015). Exploitation and application of a highly sensitive Ru(II) complex-based phosphorescent chemodosimeter for Hg^2+^ in aqueous solutions and living cells. ACS Appl. Mater. Interfaces.

[70] Dong XY (2017). Tetranuclear Zn(II) complex based on an asymmetrical salamo-type chelating ligand: synthesis, structural characterization, and fluorescence property. J. Chin. Chem. Soc..

[71] Hao J (2017). Three multinuclear Co(II), Zn(II) and Cd(II) complexes based on a single-armed salamo-type bisoxime: syntheses, structural characterizations and fluorescent properties. J. Coord. Chem..

[72] Zheng SS (2017). Four salamo-type 3d–4f hetero-bimetallic [Zn^II^Ln^III^] complexes: syntheses, crystal structures, and luminescent and magnetic properties. New J. Chem..

[73] Dong WK (2017). Luminescent properties of heterotrinuclear 3d–4f complexes constructed from a naphthalenediol-based acyclic bis(salamo)-type ligand. Spectrochim. Acta A.

[74] Li G (2017). Syntheses, crystal structures and thermal behaviors of two supramolecular salamo-type cobalt(II) and zinc(II) complexes. Crystals.

[75] Chen L (2017). Structural variation and luminescence properties of tri- and dinuclear Cu^II^ and Zn^II^ complexes constructed from a naphthalenediol-based bis(salamo)-type ligand. Cryst. Growth Des..

[76] Wang L (2017). Synthesis, crystal structure, luminescence, electrochemical and antimicrobial properties of bis(salamo)-based co(II) complex. Crystals.

[77] Hao J (2017). Four homo- and hetero-bismetallic 3d/3d-2s complexes constructed from a naphthalenediol-based acyclic bis(salamo)-type tetraoxime ligand. Polyhedron.

[78] Dong WK (2017). A reversible “turn-on” fluorescent sensor for selective detection of Zn^2+^. Sens. Actuators B..

