# Supplementary material for: Exploration and application of a highly sensitive bis(salamo)-based fluorescent sensor for B4O72− in water-containing systems and living cells
Source: Sci Rep. 2018 Sep 19;8:14058. doi: 10.1038/s41598-018-32239-y (PMC6145891; doi:10.1038/s41598-018-32239-y)
Supplement: Supplementary file 1 — Supplementary Material [file 41598_2018_32239_MOESM1_ESM.docx]

**Exploration and application of a highly sensitive bis(salamo)-based fluorescent sensor for B_4_O_7_^2-^ in water-containing systems and living cells**

**Lu-Mei Pu**^*,^**^1^, Xiao-Yan Li^2^, Jing Hao^2^, Yin-Xia Sun^2^, Yang Zhang^2^, Hai-Tao Long^1^ & Wen-Kui Dong**^*,^**^2^**

^1^ College of Science, Gansu Agricultural University, Lanzhou 730070, China. Correspondence and requests for materials should be addressed to L. M. Pu. (email: pulm@gsau.edu.cn)

^2^ School of Chemical and Biological Engineering, Lanzhou Jiaotong University, Lanzhou 730070, China. Correspondence and requests for materials should be addressed to W. K. Dong. (email: dongwk@126.com)

**Supporting Information**

**Figure Legends:**

**Figure S1.** ^1^H NMR spectra of sensor H_4_L in DMSO-*d*_6_.

**Figure S2.** (a) UV-vis spectra of sensor H_4_L (1×10^−5^ M) recorded in Tris-HCl buffer (DMF/H_2_O = 9:1, *v*/*v*, pH = 7) solutions after addition of 39.0 equiv. of various anions. (b) Absorption spectra of sensor H_4_L in Tris-HCl buffer (DMF/H_2_O = 9:1, *v*/*v*, pH = 7) solutions with gradual addition of B_4_O_7_^2-^ (0.0 to 39.0 equiv.).

**Figure S3.** ^1^H NMR spectra of sensor H_4_L and H_4_L-B_4_O_7_^2-^ in DMSO-*d*_6_.

**Figure S****4.** (a) Chelated backbones of four-coordinated organoboron compounds. (b) The process of PET recognition and blocking.

**Figure S5.** (a) Plot of fluorescence intensity at 430 nm as a function of [B_4_O_7_^2-^] / [ H_4_L] molar ratio. (b) Linear fitting of sensor H_4_L to B_4_O_7_^2-^ anion binding constant.

**Figure S6.** (a) Fluorescence intensity changes of the solution contains sensor H_4_L and 40.0 equiv. B_4_O_7_^2-^ with the delay of time. (b) Fluorescence intensity changes of the solution contains sensor H_4_L and 40.0 equiv. B_4_O_7_^2-^ at different temperatures.

**
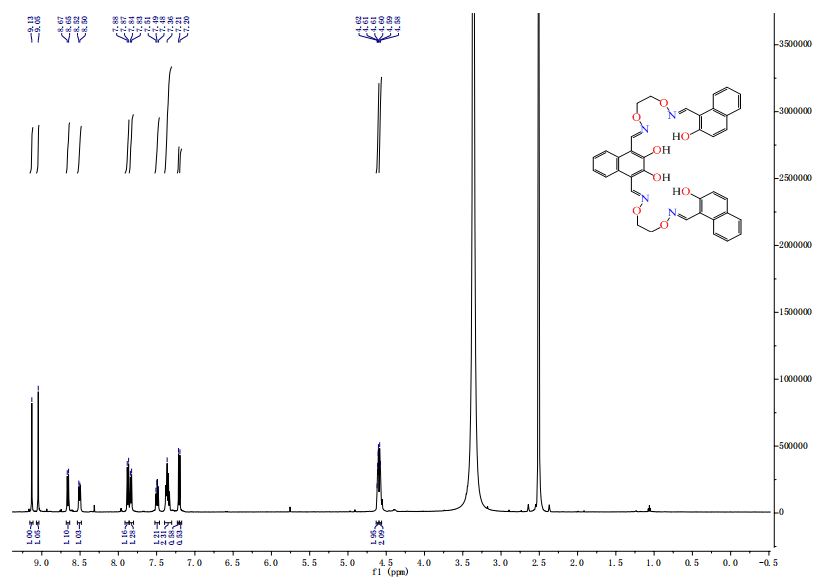
**

**Figure. S1**

**
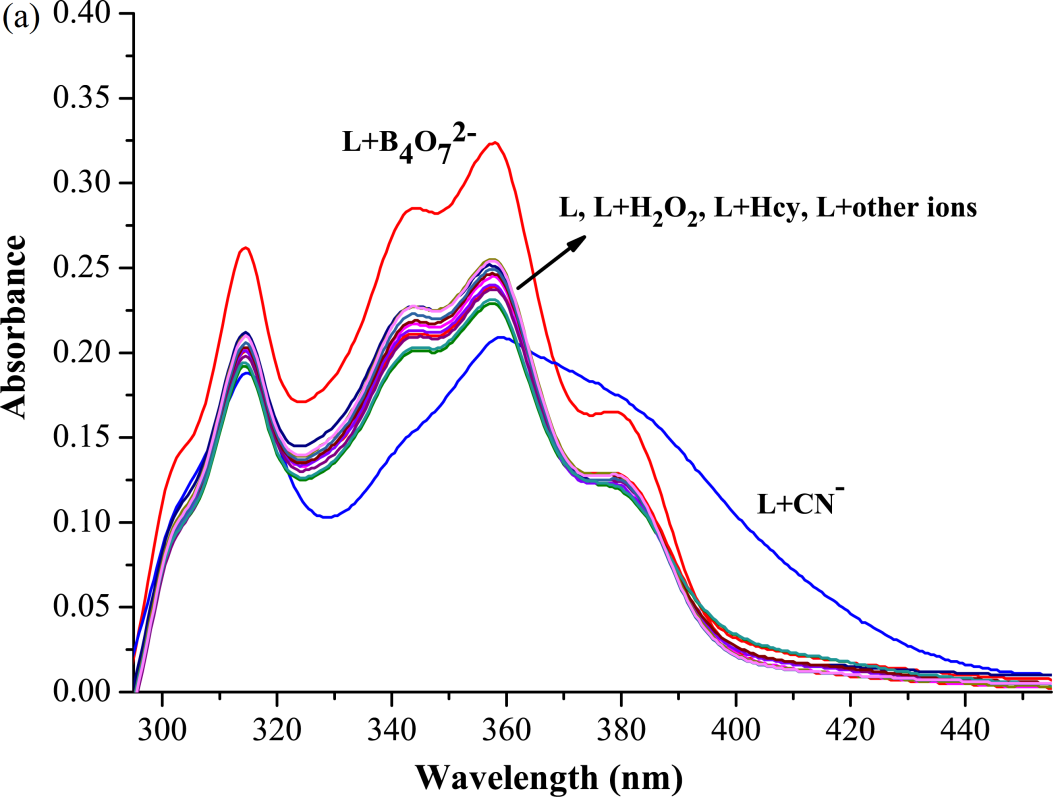
**


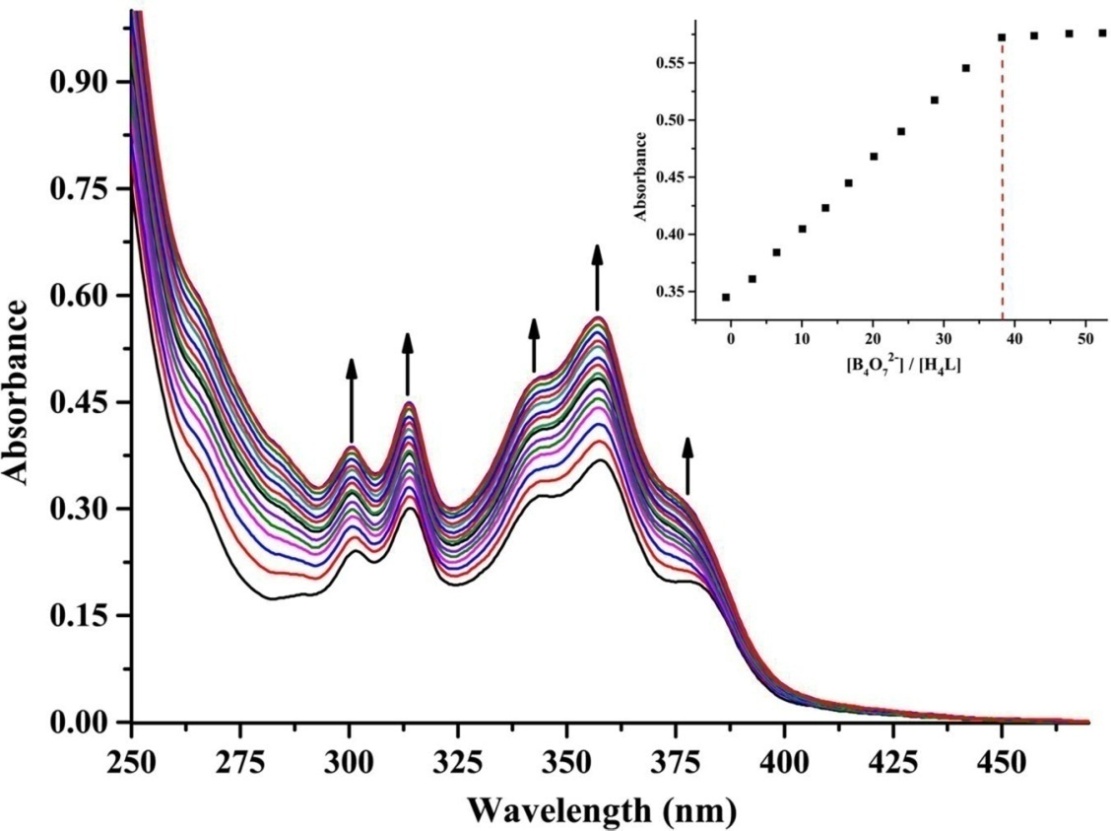


**(b)**

**Figure. S2**


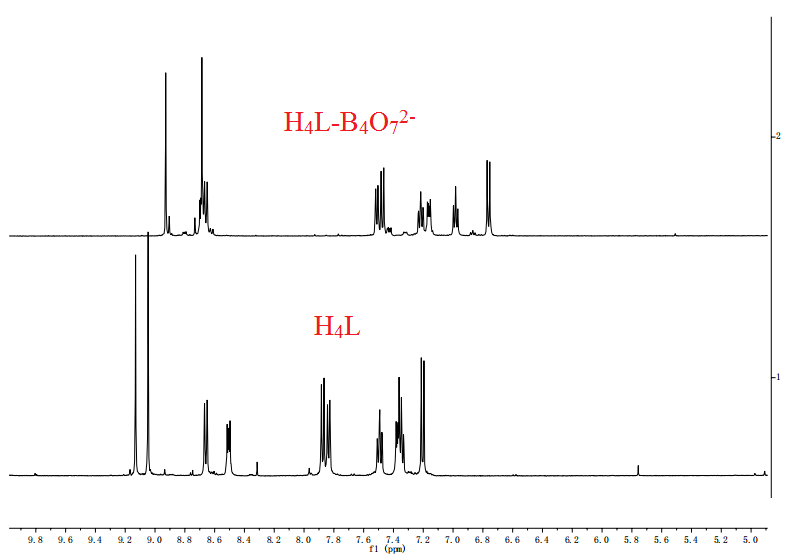


**Figure. S3**


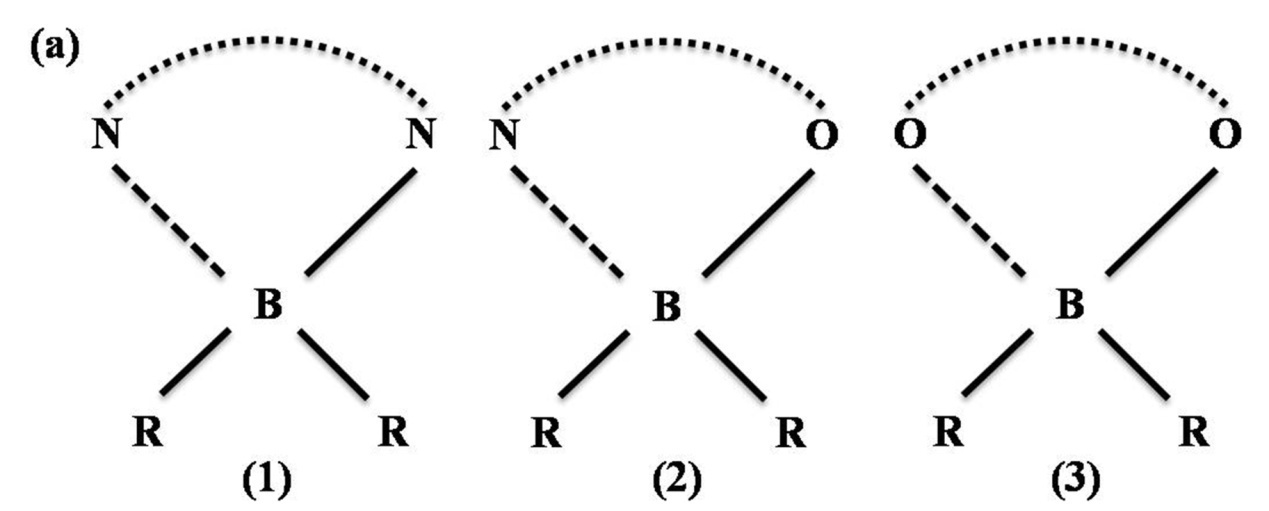


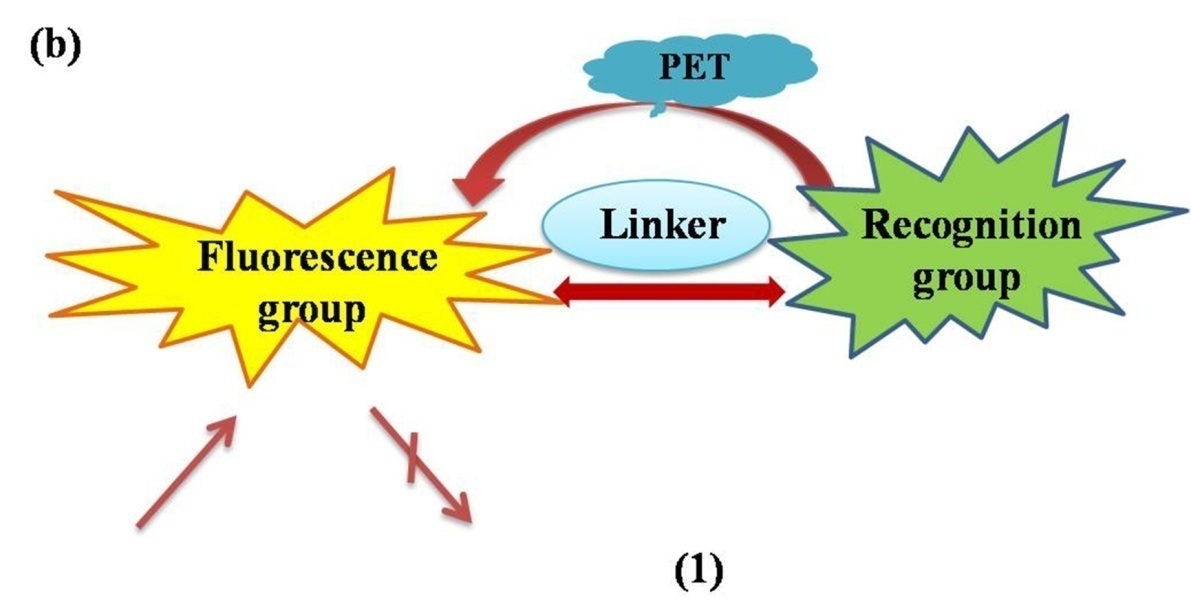


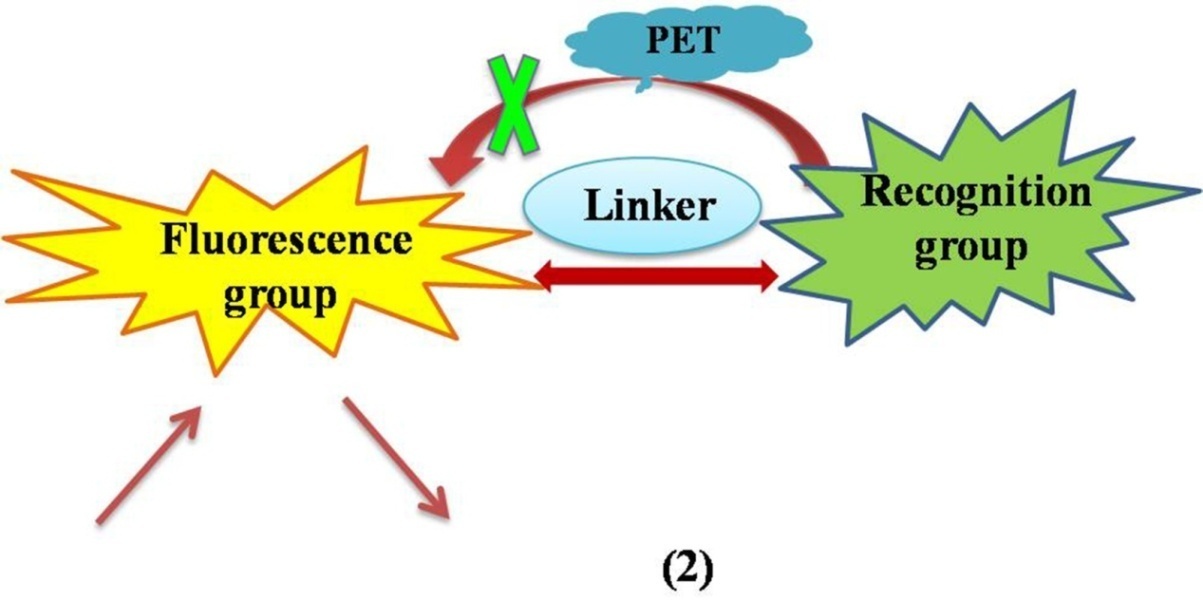


**Figure. S4**


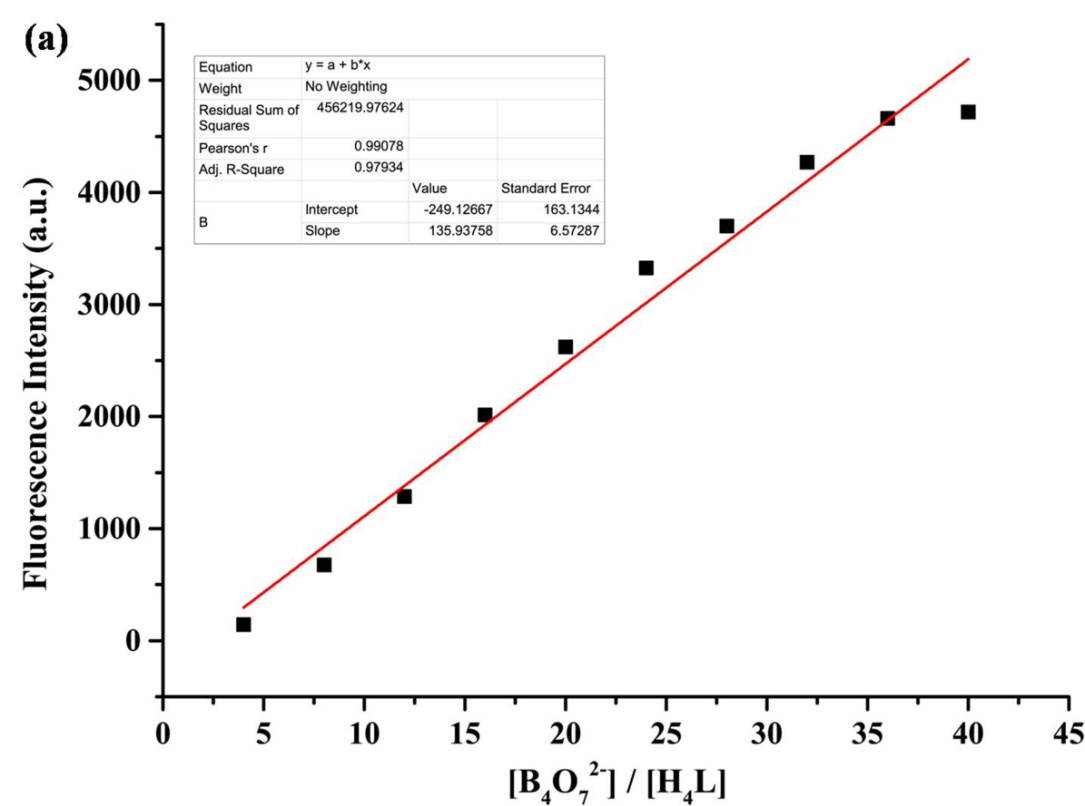


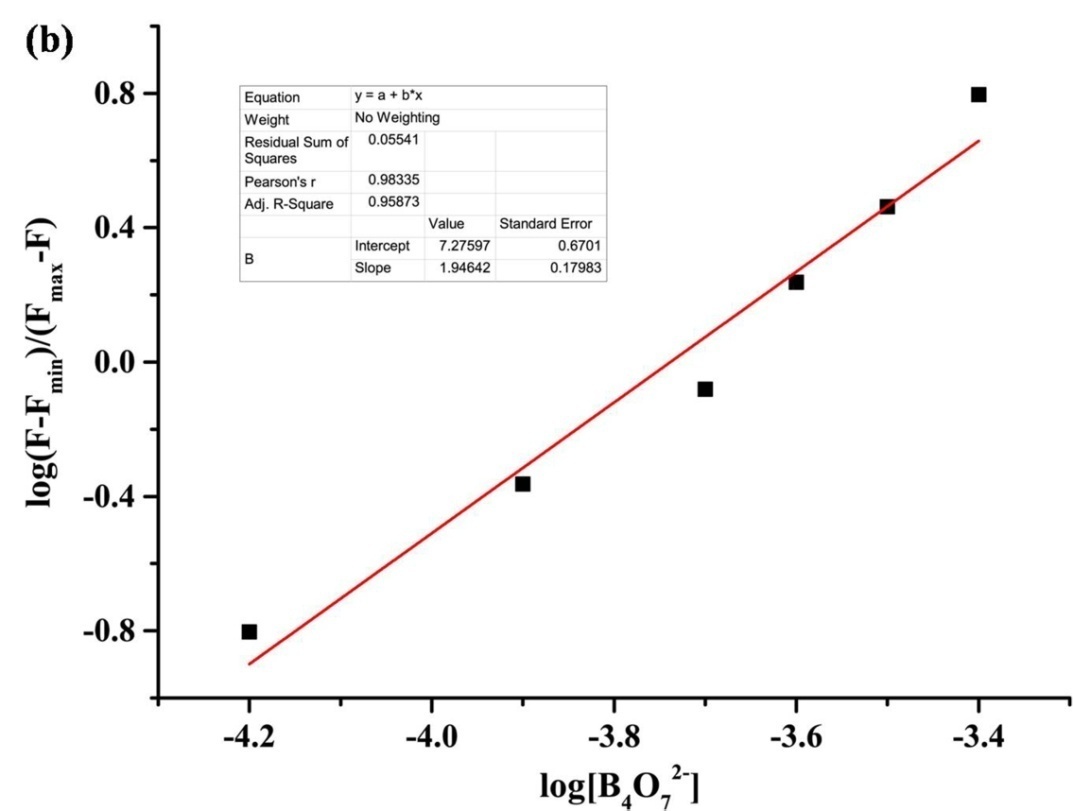


**Figure. S5**


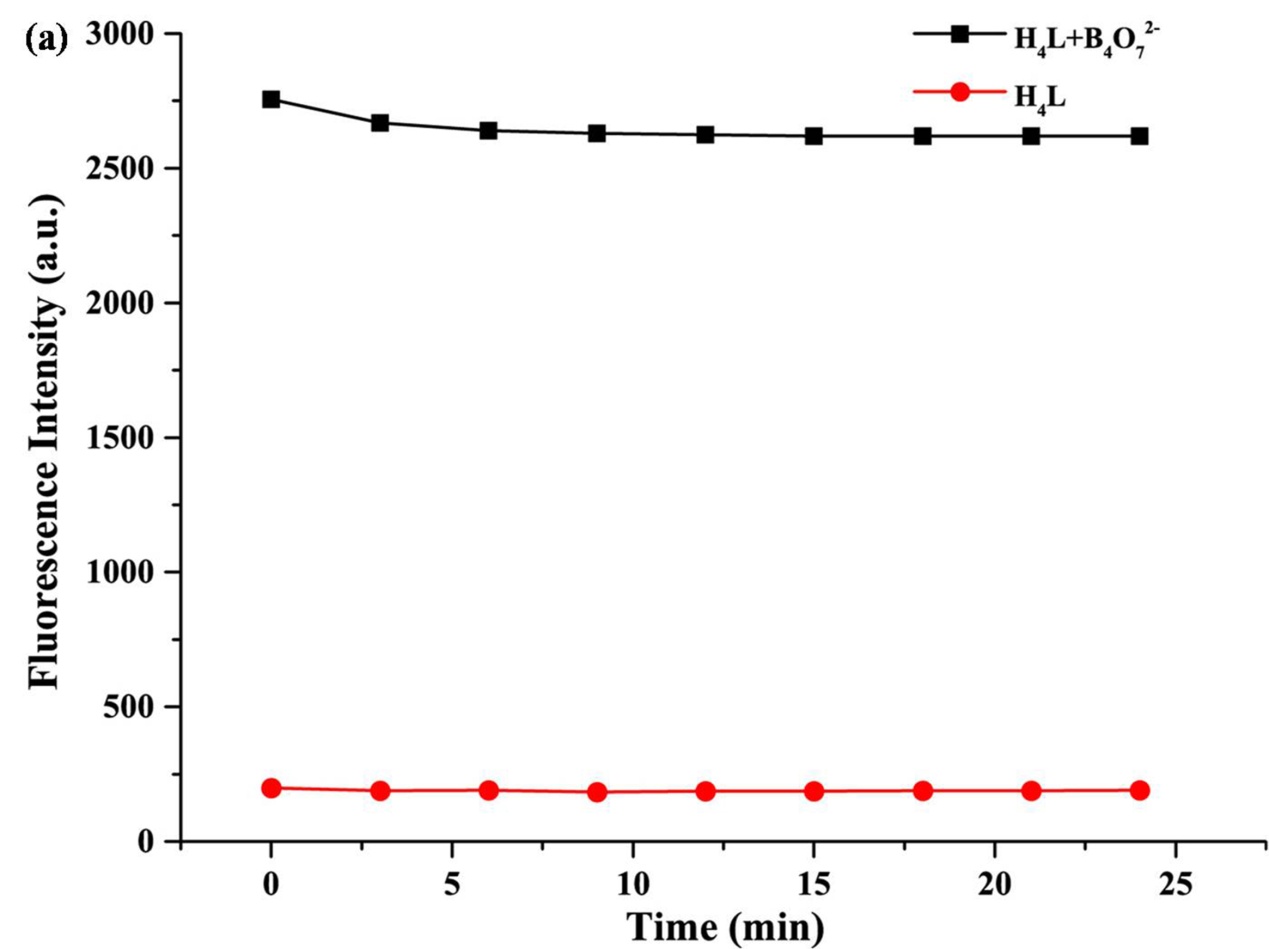


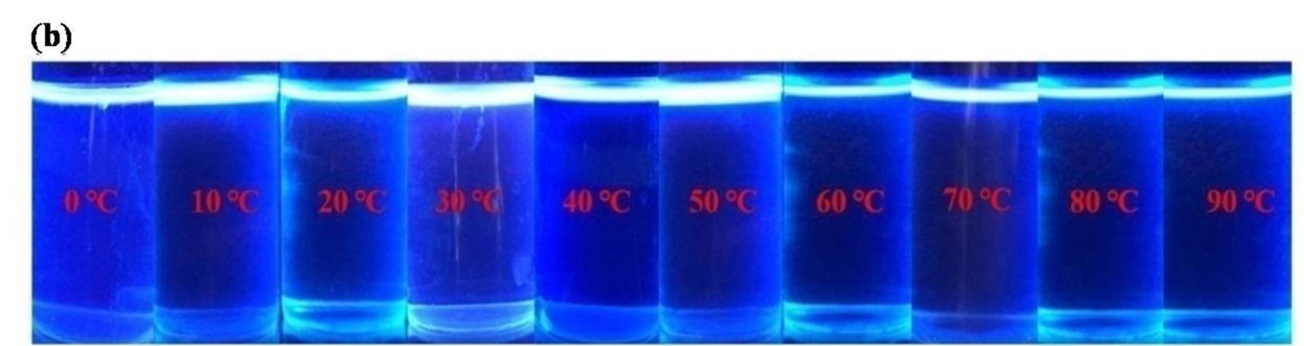


**Figure. S6**
